# Recent advances in developing disease resistance in plants

**DOI:** 10.12688/f1000research.20179.1

**Published:** 2019-11-19

**Authors:** Anuj Sharma, Jeffrey B. Jones, Frank F. White

**Affiliations:** 1 Department of Plant Pathology, University of Florida, Gainesville, FL, USA

**Keywords:** R genes, disease resistance, genome editing

## Abstract

Approaches to manipulating disease resistance in plants is expanding exponentially due to advances in our understanding of plant defense mechanisms and new tools for manipulating the plant genome. The application of effective strategies is only limited now by adoption of rapid classical genetic techniques and the acceptance of genetically engineered traits for some problems. The use of genome editing and cis-genetics, where possible, may facilitate applications that otherwise require considerable time or genetic engineering, depending on settling legal definitions of the products. Nonetheless, the variety of approaches to developing disease resistance has never been greater.

Genetic resistance represents the most economical approach to crop protection, and one goal of understanding plant/pathogen interactions at the molecular level is to facilitate disease resistance in crop species. Disease resistance is often the most dynamic component of the crop breeding process, requiring continual updating owing to pathogen adaptation to plant genotypes. An ancillary goal is to engineer resistance that is broad (effective against most or all genotypes of the pathogen) and durable (lasting through many cropping seasons). Research continues to unveil details and mechanisms that function to enable pathogens to parasitize plants and how plants defend themselves against parasitism. Increasingly, the knowledge is being implemented in strategies to enhance resistance to pathogens in crop species and to expedite resistance breeding in the field. Creative transgenic approaches continue to be explored, and the more recent genome editing tools have expanded the approaches to engineering resistance. Nonetheless, advances are needed in understanding how basic endogenous defense components work together and in generating novel resistances with components of the defense system.

Heterologous expression of pattern recognition receptors (PRRs), which recognize conserved molecules in pathogens or the products of pathogen-mediated degradation of host molecules and trigger immune responses to a wide range of pathogens, continues to be applied to a widening range of species, particularly those with recalcitrant disease issues ^1^. *Elongation Factor–Tu Receptor (EFR)* is a PRR that was identified in *Arabidopsis* and recognizes EF-Tu, a highly conserved abundant protein in prokaryotes ^2^. Many crop species apparently lack this specific or analogous receptor despite the conserved nature of EF-Tu. Transfer of *AtEFR* from *Arabidopsis* to tobacco and tomato, in particular, reduced disease severity in the field due to two bacterial pathogens with very different life styles, namely *Ralstonia solanacearum* and *Xanthomonas perforans*, the causal agents of bacterial southern wilt and bacterial spot, respectively ^3, 4^. Similar results have been reported for potato ^5^. *AtEFR* also triggered immunity in wheat upon challenge with *Pseudomonas syringae* pv. *oryzae* ^6^. However, transfer of *AtEFR* to rice did not make the lines more resistant to the most prevalent pathogen, *Xanthomonas oryzae* pv. *oryzae* (Xoo), unless the elicitor portion of EF-TU was applied prior to infection ^7, 8^. Transfer of another PRR, *Flagellin Sensitive 2 (FLS2)* from *Nicotiana,* to sweet orange reduced susceptibility to citrus canker ^9^. Use of the PRR gene *Xa21* continues to be expanded. *Xa21* of rice produces a receptor kinase that recognizes a small sulfonated peptide (RaxX) synthesized by Xoo and some related species and confers resistance to bacterial blight of rice ^10, 11^. The effectiveness of *Xa21* is limited to diseases caused by *Xanthomonas* species that produce RaxX ^10^. Fortunately, transfer of *Xa21* to banana provides resistance to bacterial wilt, which is threatening banana and enset production in east Africa, because the pathogen, *Xanthomonas vasicola* pv. *musacearum*, also makes and processes RaxX ^12^.

The application of heterologous transfer of PRRs among species will be interesting. *EFR* and *FLS2* were originally identified by the response to the isolated elicitor and do not provide complete resistance to infection due to bacterial virulence factors, which are capable of suppressing defense signaling by receptors ^13^. Perhaps it is not surprising that the introduction of heterologous PRR genes corresponding to highly conserved elicitors does not always provide protection to crop species, as their pathogens may have already adapted to defense responses elicited by infection. On the other hand, it is striking, as noted above, that some bacterial pathogens do not suppress host immunity in the presence of the PRRs. Crop species, including tomato, may have lost some PRRs in the domestication and breeding process.

The largest family of resistance (R) genes encode the nucleotide binding site and leucine-rich repeat (NBS-LRR or NLR for short) proteins. Owing to their conservation and ease of identification, NLRs are closest to what might be called industrial-scale application, and NLR mining could potentially replace R-gene introgression from related but poorer quality germplasm and crosses with related species (wide crosses). NLR members (and the associated components, which often provide the pathogen recognition function) generally provide resistance against a specific subset of pathogens, or races, that express specific effector proteins, and the NLR complex often acts in a gene-for-gene manner. More problematic is that the genes tend to function only within closely related species, possibly because of the adaptation to other components of the specific NLR complex. An early example was the transfer of the *Bs2* gene for resistance to bacterial spot disease in pepper to tomato, which suffers disease from related pathogens ^4, 14^. The NLR gene *RGA2* for resistance to *Fusarium* was transferred from a resistant diploid banana species to Cavendish banana ^15^. Another example is the transfer of an NLR from pigeon pea to soybean for resistance to soybean rust ^16^. The success of transferring NLR genes between species has led to more extensive efforts of extracting NLR homologs (often referred to as R gene analogs or RGAs) from resistant species. R gene enrichment sequencing, or RenSeq, is a sequence capture technique for the enrichment of NLR sequences ^17^. The underlying goal is to recover NLR family members from plants with known resistance and transfer candidates to the desired variety. Several variations of this method have been reported, including chemical mutagenesis of a line followed by sequence capture and association genetics of wild populations followed by sequence capture ^18, 19^. NLR capture from related species will facilitate the stacking of R genes against common core effectors of all extant pathogen genotypes and, consequentially, provide broad resistance. For example, the *Bs2* gene, which targets the ubiquitous AvrBs2 effector of *Xanthomonas*, can be combined with *Roq1*, a new R gene from *Nicotiana* directed at the common effector XopQ, and other as-yet-unidentified NLRs directed toward other conserved effectors as identified in sequencing of large strain collections from infected plants ^20, 21^. Advances in NLR gene mining and gene transfer may have come none too soon and can be applied to wheat blast outbreaks ^22^. Advancements in NLR applications will come from new methods to identify novel R genes in existing NLR libraries.

Research is also providing clues that promise to broaden the application of NLR resistance strategies and even the promise of generating NLR libraries with novel pathogen recognition properties *de novo*. Single NLR-related R gene transfer between distantly related species often fails, and broader application of NLRs will come from a greater understanding of NLR function. NLR-like R genes are simply the variable genetic component of NLR signaling complexes, which occur in a variety of forms. The complex can include a guarded protein, alternatively called guardee or sensor, and the sensor may be an integrated domain of the NLR ^23^. The guardee may be a defense signaling protein or other component that is targeted by pathogens to enhance susceptibility. In some cases, the guardee has no apparent function other than to recognize the effector or effector activity of the pathogen. In the latter case, the guardee is referred to as a decoy. In a variety of cases, the components consist of a pair of NLR or NLR-like genes ^24^. The NLRs *RPS4* and *RRS1*, for example, are R genes from *Arabidopsis* and provide resistance against the bacteria *Pseudomonas syringae* and *Ralstonia solanacearum*, respectively. The RPS4/RRS1 pair is a remarkable case where the variation between resistance and susceptibility toward each of the two pathogens was based on separate components. However, both genes are, in fact, required for the resistance to each pathogen ^25^. Furthermore, tomato plants expressing both *RPS4* and *RRS1* were resistant to both bacteria, and similar results were obtained for transgenic cucumber expressing *RPS4/RRS1* against anthracnose ^26^. Manipulation of the complexes may also allow changing the complex to recognize other pathogens. In the *Arabidopsis*– *P. syringae* system, the R gene *PBS1* product is perceived as a decoy working in concert with the NLR *RPS5* and cleaved by the secreted bacterial protease effector AvrPphB. The cleavage of *PSB1* is recognized by R-gene *RPS5*, resulting in the detection of bacteria and a resistance reaction ^27^. A novel *PBS1* gene was created by substituting the cleavage site with cleavage site sequences that are recognized by other viral and secreted bacterial proteases ^28^. When expressed together, the modified *PBS1* genes conferred *RPS5*-mediated resistance to new pathogens. Changes in the integrated sensor domain of the Pik group of NLRs directed at the rice blast pathogen effectors indicate that manipulation of the domain could produce new recognition motifs ^29^. In the potato– *Phytophthora infestans* system, the resistance gene *R3a* is activated by the RXLR effector, AVR3a ^KI^, but not by the allelic product AVR3a ^EM^, and, through random mutagenesis, a mutant version of R3a was identified that recognized AVR3a ^EM^ ^30^. Interestingly, mutation in the same position in the *I2* gene, a homologue of the *R3a* gene in tomato, made the gene more responsive to AVR3a and conferred partial resistance to *P. infestans* and expanded the gene’s effectiveness to an additional fungal pathogen ^31^. Recent studies of the structural changes that the NLR proteins themselves undergo upon elicitation may also provide insight for improved manipulation of this effector class ^32, 33^. Further utility of the NLR class will come from improved structural models and associated components and induced variation by gene targeting strategies ^23^.

Ectopic expression of defense-related, toxin, and other miscellaneous genes has always been a major part of the toolbox in engineering resistance. The approaches have been applied to recalcitrant disease problems and, in a variety of cases, have reached or completed confined field trial stage ^34^. Secreted anti-microbial peptides (AMPs) are used in a variety of crop species ^35^. Cecropin is an AMP naturally produced by a moth, *Hyalophora cecropia*, that confers a broad spectrum of protection against a wide range of pathogens. Rice seed expressing cecropin A from endosperm-specific promoter exhibited resistance to infection by *Fusarium verticillioides* and *Dickeya dadantii* ^36^. A synthetic version of cecropin expressed in citrus was reported to be effective against the bacterium *Candidatus Liberibacter asiaticus*, the causal agent of huanglongbing (HLB) ^37^ *.* Expression of *de novo* designed AMP SP1-1 in tomato fused with secretion of the signal from radish defensin provided resistance to bacterial spot ^38^. Using pathogen physiology against itself has also provided promising results. Diffusible signal factor (DSF) is a mobile extracellular signal molecule produced by bacterial pathogens which controls cell density-dependent patterns of gene expression. In *Xylella fastidiosa*, DSF production is conferred by the gene *rpfF*. Ectopic production of DSF results in hypervirulence but decreases transmissibility by vectors owing to interference with bacterial gene regulation ^39^. Susceptible scion grafted to transgenic rootstock also displayed resistance to the pathogen in field trials. Similar reductions in disease severity were also observed by ectopic expression of *rpfF* in citrus and tobacco ^40^.

Strides in genome editing, particularly the CRISPR–Cas9 system, have increased interest towards the development of disease resistance through the modification of susceptible (S) genes of the plant ^41^. The classic example is *mlo*, a recessive R gene of barley with resistance toward powdery mildew ^42^. The null allele provides broad, durable resistance against the pathogen *Blumeria graminis* f. sp. *hordei*. Simultaneous editing of all three homoeoalleles of *MLO* locus in hexaploid wheat conferred recessive resistance against powdery mildew in one generation ^43^. Disruption of *downy mildew resistance-6 (DMR6*) was originally identified in *Arabidopsis* and suppresses free salicylic acid (SA) levels ^44^. Enhanced SA levels are associated with reduced susceptibility to a variety of pathogens, particularly bacterial pathogens. Mutations created by CRISPR–Cas9 in *DMR6* orthologs in tomato were reported to confer resistance to a number of pathogens, including *P. syringae* pv. *tomato*, *Phytophthora capsica, X. perforans*, and *Xanthomonas gardneri* ^45^. Modifications of the SA pathway adds to the widespread experimental utilization of ectopic expression of the SA receptor *nonexpressor of pathogenesis related genes 1* ( *NPR1*), which has been reviewed extensively ^46, 47^.

Diseases that involve transcription activator-like effectors (TALes) are excellent candidates for genome editing. TALes, which are deployed by many members of the bacterial genus *Xanthomonas*, function by binding to specific DNA sequences, known as effector binding elements (EBEs), in the promoters of S genes and promote heroic levels of S gene expression and, consequently through S gene product function, enhance disease susceptibility. Polymorphisms in EBEs by preventing TALe binding can provide recessive resistance in cases where TALes play a critical role in disease development. The citrus gene *CsLOB1* is a susceptibility factor for citrus canker induced by the TALe PthA4 of *Xanthomonas citri*. CRISPR–Cas9-mediated modification in the EBE in the promoter region of *CsLOB1* improved canker resistance and reduced canker development in sweet orange and grapefruit ^48– 50^. Bacterial blight of rice is highly dependent on TALe-mediated expression of sucrose transporters known as SWEET genes. Three SWEET genes are known to be targeted by strains of Xoo, and natural polymorphisms occur in the SWEET gene promoters that provide recessive resistance to some strains of the pathogen Xoo. *OsSWEET14* is an S gene in rice for infections by Xoo. Mutations in the promoter EBE region for the TALe effectors AvrXa7 and PthXo3 using TALe nucleases (TALENs) led to loss of expression of S gene and resistance to strains depending on those two TALes for virulence ^51– 53^. The S gene *OsSWEET13* is upregulated by TALes, leading to bacterial blight ^54^. Modification of the promotor of *OsSWEET13* rice by CRISPR–Cas9 treatment resulted in resistance against strains that require *OsSWEET13* expression ^54^. Modification of all three genes simultaneously has been accomplished with CRISPR–Cas9 and provides broad resistance against extant strains ^55^.

Disruption of S genes, by either editing or silencing, potentially comes with its own set of problems. First, these resistances are recessive, requiring homozygous mutant plants to screen for resistance. Second, these genes often have functional roles in the plant, and deletion or modification of expression may result in growth defects and/or loss of yield ^56^. For example, a CRISPR–Cas9-mediated complete disruption of the *OsSEC3A* gene in rice caused enhanced defense response against blast caused by *Magnaporthe oryzae*. However, the resistant plant showed dwarf stature and lesion-mimic phenotype ^57, 58^. Similarly, the mutations in wheat *MLO* conferred resistance against powdery mildew, but some mutants have been reported to have a leaf chlorosis phenotype similar to what is sometimes observed in barley ^43, 59^.

The affinity of TALes to bind to specific EBE sequences can be hijacked for activation of resistance by TALes. R gene promoters have been modified to include a concatemer of EBE sites for TALes from multiple strains of a pathogen ^60, 61^. TAL EBEs can also be added to promoters to recruit TALe-mediated expression of autonomous R genes or avirulence proteins leading to resistance reactions ^62, 63^.

Because of their dependence on host functions, viruses are vulnerable to manipulations of host genes. Eukaryotic translation initiation factors (eIF4E) are essential for infection by single-stranded positive-sense RNA viruses ^64^. They are also vital for normal initiation of translation in host cells. Due to the presence of two isoforms, null mutants of any one isoform can result in resistance to a number of viruses ^65, 66^. However, simultaneous knockout of both isoforms can lead to lethality or impaired growth ^67^. Genome editing can be used to create novel functional alleles by mimicking natural variation of eIF4E in resistant plant species ^68^. These synthetic genes for elF4E confer resistance to viruses without affecting plant physiology.

Site-specific nucleases have also been deployed as functional components in plants for resistance against viruses. This approach imitates the viral immunity in prokaryotes in which the CRISPR system recognizes and cleaves the viral genome *in vivo* ^69^. The CRISPR–Cas9 system expressed transiently in *Nicotiana* along with sgRNA that recognize the viral genome significantly reduced geminivirus accumulation not just in inoculated areas but also systemically ^70, 71^. The ability of viruses to overcome immunity is much lower when targeting intergenic regions compared to coding regions ^57^. A powerful system to control multiple viruses in cotton leaf curl disease related to begomovirus complex can be achieved by multiplex CRISPR system, imitating CRISPR function in bacteria ^72^.

The analyses of naturally occurring resistances, particularly cases of single gene broad R genes, has provided a remarkable variety of genes beyond the now-classic PRRs and NLRs. Wheat breeding and characterization of many wheat relatives has provided many broad, durable R gene candidates, and the characterization of a number of rust R genes of wheat warns us that knowledge of broad, durable resistance will likely come with considerable advances in our understanding of plant physiology. The broad R genes, *Lr34* and *Lr67,* provide broad partial resistance to wheat leaf rust ( *Puccinia triticina*) and to other pathogens, for example, yellow rust ( *Puccinia striiformis* f. sp. *tritici*). The two genes were discovered to encode ABC-type and hexose transporters, respectively ^73, 74^. Despite the novel properties of the gene products, transfer of the genes, at least within the cereal family, indicates that the genes can function similarly in related species ^75– 79^. *Lr67* appears to be impaired in sugar transport, while *Lr34* product was shown recently to be capable of transporting abscisic acid (ABA) ^80^. Whether these attributes are relevant to the broad and multi-pathogen resistance is unknown. At the same time, the functionality in related species indicates a conserved function.

Success of many of the advances in engineering disease resistance in crop species, of course, depends on societal acceptance of various approaches to plant genome modification. Ectopic expression of heterologous transgenes or silencing of genes generally comes under the guise of foreign DNA transfer and still faces considerable headwinds owing to acceptance and regulatory protocols. Modification of transgenic classifications, for example, the concept of cisgenics (allowing the addition of genes from a crossable species) as opposed to transgenics (the addition of a gene or genes from a non-crossable species), may increase the workable space in crop modification ^81^. Genome modification using site-specific nucleases and removal of vector sequences by crossing, or use of vector-free approaches, has been accepted in some agencies not requiring regulation as a transgenic event. However, acceptance has not been universally accepted. Regardless of regulatory issues, our understanding of plant resistance mechanisms has increased considerably in the last five years, and new insights into defenses against resistance that incorporate abiotic and other physiological pathways of plants will undoubtedly be forthcoming. The discoveries will inform mutational and traditional breeding strategies in the absence of adoption of gene transfer or gene editing technologies and help meet the future needs for food output for a growing world population and climate-challenged food production system.

## References

[1] Tang D Wang G Zhou JM: Receptor Kinases in Plant-Pathogen Interactions: More Than Pattern Recognition. *Plant Cell.* 2017;29(4):618–37. 10.1105/tpc.16.00891 28302675 PMC5435430

[2] Zipfel C Kunze G Chinchilla D: Perception of the bacterial PAMP EF-Tu by the receptor EFR restricts *Agrobacterium*-mediated transformation. *Cell.* 2006;125(4):749–60. 10.1016/j.cell.2006.03.037 16713565

[3] Lacombe S Rougon-Cardoso A Sherwood E: Interfamily transfer of a plant pattern-recognition receptor confers broad-spectrum bacterial resistance. *Nat Biotechnol.* 2010;28(4):365–9. 10.1038/nbt.1613 20231819

[4] Kunwar S Iriarte F Fan Q: Transgenic Expression of *EFR* and *Bs2* Genes for Field Management of Bacterial Wilt and Bacterial Spot of Tomato. *Phytopathology.* 2018;108(12):1402–11. 10.1094/PHYTO-12-17-0424-R 29923802

[5] Boschi F Schvartzman C Murchio S: Enhanced Bacterial Wilt Resistance in Potato Through Expression of *Arabidopsis* EFR and Introgression of Quantitative Resistance from *Solanum commersonii*. *Front Plant Sci.* 2017;8:1642. 10.3389/fpls.2017.01642 29033958 PMC5627020

[6] Schoonbeek HJ Wang HH Stefanato FL: Arabidopsis EF-Tu receptor enhances bacterial disease resistance in transgenic wheat. *New Phytol.* 2015;206(2):606–13. 10.1111/nph.13356 25760815

[7] Lu F Wang H Wang S: Enhancement of innate immune system in monocot rice by transferring the dicotyledonous elongation factor Tu receptor EFR. *J Integr Plant Biol.* 2015;57(7):641–52. 10.1111/jipb.12306 25358295

[8] Schwessinger B Bahar O Thomas N: Transgenic expression of the dicotyledonous pattern recognition receptor EFR in rice leads to ligand-dependent activation of defense responses. *PLoS Pathog.* 2015;11(3):e1004809. 10.1371/journal.ppat.1004809 25821973 PMC4379099

[9] Hao G Pitino M Duan Y: Reduced Susceptibility to *Xanthomonas citri* in Transgenic Citrus Expressing the FLS2 Receptor From *Nicotiana benthamiana*. *Mol Plant Microbe Interact.* 2016;29(2):132–42. 10.1094/MPMI-09-15-0211-R 26554734

[10] Liu F McDonald M Schwessinger B: Variation and inheritance of the *Xanthomonas raxX-raxSTAB* gene cluster required for activation of XA21-mediated immunity. *Mol Plant Pathol.* 2019;20(5):656–72. 10.1111/mpp.12783 30773771 PMC6637879

[11] Pruitt RN Joe A Zhang W: A microbially derived tyrosine-sulfated peptide mimics a plant peptide hormone. *New Phytol.* 2017;215(2):725–36. 10.1111/nph.14609 28556915 PMC5901733

[12] Tripathi JN Lorenzen J Bahar O: Transgenic expression of the rice *Xa21* pattern-recognition receptor in banana ( *Musa sp*.) confers resistance to *Xanthomonas campestris pv. musacearum*. *Plant Biotechnol J.* 2014;12(6):663–73. 10.1111/pbi.12170 24612254 PMC4110157

[13] Macho AP Zipfel C: Targeting of plant pattern recognition receptor-triggered immunity by bacterial type-III secretion system effectors. *Curr Opin Microbiol.* 2015;23:14–22. 10.1016/j.mib.2014.10.009 25461568

[14] Horvath DM Pauly MH Hutton SF: The pepper BS2 gene confers effective field resistance to bacterial leaf spot and yield enhancement in Florida tomatoes. *Acta Hortic.* 2015;47–51. 10.17660/ActaHortic.2015.1069.5

[15] Dale J James A Paul JY: Transgenic Cavendish bananas with resistance to Fusarium wilt tropical race 4. *Nat Commun.* 2017;8(1):1496. 10.1038/s41467-017-01670-6 29133817 PMC5684404

[16] Kawashima CG Guimarães GA Nogueira SR: A pigeonpea gene confers resistance to Asian soybean rust in soybean. *Nat Biotechnol.* 2016;34(6):661–5. 10.1038/nbt.3554 27111723

[17] Jupe F Witek K Verweij W: Resistance gene enrichment sequencing (RenSeq) enables reannotation of the NB-LRR gene family from sequenced plant genomes and rapid mapping of resistance loci in segregating populations. *Plant J.* 2013;76(3):530–44. 10.1111/tpj.12307 23937694 PMC3935411

[18] Arora S Steuernagel B Gaurav K: Resistance gene cloning from a wild crop relative by sequence capture and association genetics. *Nat Biotechnol.* 2019;37(2):139–43. 10.1038/s41587-018-0007-9 30718880

[19] Steuernagel B Periyannan SK Hernández-Pinzón I: Rapid cloning of disease-resistance genes in plants using mutagenesis and sequence capture. *Nat Biotechnol.* 2016;34(6):652–5. 10.1038/nbt.3543 27111722

[20] Schultink A Qi T Lee A: Roq1 mediates recognition of the Xanthomonas and Pseudomonas effector proteins XopQ and HopQ1. *Plant J.* 2017;92(5):787–95. 10.1111/tpj.13715 28891100

[21] Timilsina S Abrahamian P Potnis N: Analysis of Sequenced Genomes of *Xanthomonas perforans* Identifies Candidate Targets for Resistance Breeding in Tomato. *Phytopathology.* 2016;106(10):1097–104. 10.1094/PHYTO-03-16-0119-FI 27392180

[22] Kamoun S Talbot NJ Islam MT: Plant health emergencies demand open science: Tackling a cereal killer on the run. *PLoS Biol.* 2019;17(6):e3000302. 10.1371/journal.pbio.3000302 31158224 PMC6564034

[23] Adachi H Derevnina L Kamoun S: NLR singletons, pairs, and networks: evolution, assembly, and regulation of the intracellular immunoreceptor circuitry of plants. *Curr Opin Plant Biol.* 2019;50:121–31. 10.1016/j.pbi.2019.04.007 31154077

[24] Cesari S Bernoux M Moncuquet P: A novel conserved mechanism for plant NLR protein pairs: the "integrated decoy" hypothesis. *Front Plant Sci.* 2014;5:606. 10.3389/fpls.2014.00606 25506347 PMC4246468

[25] Narusaka M Shirasu K Noutoshi Y: *RRS1* and *RPS4* provide a dual *Resistance*-gene system against fungal and bacterial pathogens. *Plant J.* 2009;60(2):218–26. 10.1111/j.1365-313X.2009.03949.x 19519800

[26] Narusaka M Kubo Y Hatakeyama K: Interfamily transfer of dual NB-LRR genes confers resistance to multiple pathogens. *PLoS One.* 2013;8(2):e55954. 10.1371/journal.pone.0055954 23437080 PMC3577827

[27] Ade J DeYoung BJ Golstein C: Indirect activation of a plant nucleotide binding site-leucine-rich repeat protein by a bacterial protease. *Proc Natl Acad Sci U S A.* 2007;104(7):2531–6. 10.1073/pnas.0608779104 17277084 PMC1790868

[28] Kim SH Qi D Ashfield T: Using decoys to expand the recognition specificity of a plant disease resistance protein. *Science.* 2016;351(6274):684–7. 10.1126/science.aad3436 26912853

[29] De la Concepcion JC Franceschetti M Maqbool A: Polymorphic residues in rice NLRs expand binding and response to effectors of the blast pathogen. *Nat Plants.* 2018;4(8):576–85. 10.1038/s41477-018-0194-x 29988155

[30] Chapman S Stevens LJ Boevink PC: Detection of the virulent form of AVR3a from *Phytophthora infestans* following artificial evolution of potato resistance gene *R3a*. *PLoS One.* 2014;9(10):e110158. 10.1371/journal.pone.0110158 25340613 PMC4207746

[31] Giannakopoulou A Steele JF Segretin ME: Tomato I2 Immune Receptor Can Be Engineered to Confer Partial Resistance to the Oomycete *Phytophthora infestans* in Addition to the Fungus *Fusarium oxysporum*. *Mol Plant Microbe Interact.* 2015;28(12):1316–29. 10.1094/MPMI-07-15-0147-R 26367241

[32] Wang J Wang J Hu M: Ligand-triggered allosteric ADP release primes a plant NLR complex. *Science.* 2019;364(6435). pii: eaav5868. 10.1126/science.aav5868 30948526

[33] Wang J Hu M Wang J: Reconstitution and structure of a plant NLR resistosome conferring immunity. *Science.* 2019;364(6435): pii: eaav5870 10.1126/science.aav5870 30948527

[34] Tripathi L Atkinson H Roderick H: Genetically engineered bananas resistant to Xanthomonas wilt disease and nematodes. *Food Energy Secur.* 2017;6(2):37–47. 10.1002/fes3.101 28713567 PMC5488630

[35] Breen S Solomon PS Bedon F: Surveying the potential of secreted antimicrobial peptides to enhance plant disease resistance. *Front Plant Sci.* 2015;6:900. 10.3389/fpls.2015.00900 26579150 PMC4621407

[36] Bundó M Montesinos L Izquierdo E: Production of cecropin A antimicrobial peptide in rice seed endosperm. *BMC Plant Biol.* 2014;14:102. 10.1186/1471-2229-14-102 24755305 PMC4032361

[37] Zou X Jiang X Xu L: Transgenic citrus expressing synthesized *cecropin* B genes in the phloem exhibits decreased susceptibility to Huanglongbing. *Plant Mol Biol.* 2017;93(4–5):341–53. 10.1007/s11103-016-0565-5 27866312

[38] Herrera Diaz A Kovacs I Lindermayr C: Inducible Expression of the *De-Novo* Designed Antimicrobial Peptide SP1-1 in Tomato Confers Resistance to *Xanthomonas campestris* pv. *vesicatoria*. *PLoS One.* 2016;11(10):e0164097. 10.1371/journal.pone.0164097 27706237 PMC5051901

[39] Lindow S Newman K Chatterjee S: Production of *Xylella fastidiosa* diffusible signal factor in transgenic grape causes pathogen confusion and reduction in severity of Pierce's disease. *Mol Plant Microbe Interact.* 2014;27(3):244–54. 10.1094/MPMI-07-13-0197-FI 24499029

[40] Caserta R Souza-Neto RR Takita MA: Ectopic Expression of *Xylella fastidiosa rpfF* Conferring Production of Diffusible Signal Factor in Transgenic Tobacco and Citrus Alters Pathogen Behavior and Reduces Disease Severity. *Mol Plant Microbe Interact.* 2017;30(11):866–75. 10.1094/MPMI-07-17-0167-R 28777044

[41] Borrelli VMG Brambilla V Rogowsky P: The Enhancement of Plant Disease Resistance Using CRISPR/Cas9 Technology. *Front Plant Sci.* 2018;9:1245. 10.3389/fpls.2018.01245 30197654 PMC6117396

[42] Kusch S Panstruga R: *mlo*-Based Resistance: An Apparently Universal "Weapon" to Defeat Powdery Mildew Disease. *Mol Plant Microbe Interact.* 2017;30(3):179–89. 10.1094/MPMI-12-16-0255-CR 28095124

[43] Wang Y Cheng X Shan Q: Simultaneous editing of three homoeoalleles in hexaploid bread wheat confers heritable resistance to powdery mildew. *Nat Biotechnol.* 2014;32(9):947–51. 10.1038/nbt.2969 25038773

[44] Zeilmaker T Ludwig NR Elberse J: DOWNY MILDEW RESISTANT 6 and DMR6-LIKE OXYGENASE 1 are partially redundant but distinct suppressors of immunity in Arabidopsis. *Plant J.* 2015;81(2):210–22. 10.1111/tpj.12719 25376907

[45] de Toledo Thomazella DP Brail Q Dahlbeck D: CRISPR-Cas9 mediated mutagenesis of a *DMR6* ortholog in tomato confers broad-spectrum disease resistance. *bioRxiv.* 2016 10.1101/064824 PMC8271637 34215692

[46] Xu G Yuan M Ai C: uORF-mediated translation allows engineered plant disease resistance without fitness costs. *Nature.* 2017;545(7655):491–4. 10.1038/nature22372 28514448 PMC5532539

[47] Silva KJP Mahna N Mou Z: *NPR1* as a transgenic crop protection strategy in horticultural species. *Hortic Res.* 2018;5: 15. 10.1038/s41438-018-0026-1 29581883 PMC5862871

[48] Jia H Zhang Y Orbović V: Genome editing of the disease susceptibility gene *CsLOB1* in citrus confers resistance to citrus canker. *Plant Biotechnol J.* 2017;15(7):817–23. 10.1111/pbi.12677 27936512 PMC5466436

[49] Jia H Orbovic V Jones JB: Modification of the PthA4 effector binding elements in Type I CsLOB1 promoter using Cas9/sgRNA to produce transgenic Duncan grapefruit alleviating XccΔpthA4:dCsLOB1.3 infection. *Plant Biotechnol J.* 2016;14(5):1291–301. 10.1111/pbi.12495 27071672 PMC11389130

[50] Peng A Chen S Lei T: Engineering canker-resistant plants through CRISPR/Cas9-targeted editing of the susceptibility gene *CsLOB1* promoter in citrus. *Plant Biotechnol J.* 2017;15(12):1509–19. 10.1111/pbi.12733 28371200 PMC5698050

[51] Li T Liu B Spalding MH: High-efficiency TALEN-based gene editing produces disease-resistant rice. *Nat Biotechnol.* 2012;30(5):390–2. 10.1038/nbt.2199 22565958

[52] Cao H Li X Dong X: Generation of broad-spectrum disease resistance by overexpression of an essential regulatory gene in systemic acquired resistance. *Proc Natl Acad Sci U S A.* 1998;95(11):6531–6. 10.1073/pnas.95.11.6531 9601001 PMC34547

[53] Blanvillain-Baufumé S Reschke M Solé M: Targeted promoter editing for rice resistance to *Xanthomonas oryzae* pv. *oryzae* reveals differential activities for *SWEET14*-inducing TAL effectors. *Plant Biotechnol J.* 2017;15(3):306–17. 10.1111/pbi.12613 27539813 PMC5316920

[54] Zhou J Peng Z Long J: Gene targeting by the TAL effector PthXo2 reveals cryptic resistance gene for bacterial blight of rice. *Plant J.* 2015;82(4):632–43. 10.1111/tpj.12838 25824104

[55] Oliva R Ji C Atienza-Grande G: Broad-spectrum resistance to bacterial blight in rice using genome editing. *Nat Biotechnol.* 2019. 10.1038/s41587-019-0267-z PMC6831514 31659337

[56] Zaidi SS Mukhtar MS Mansoor S: Genome Editing: Targeting Susceptibility Genes for Plant Disease Resistance. *Trends Biotechnol.* 2018;36(9):898–906. 10.1016/j.tibtech.2018.04.005 29752192

[57] Ali Z Zaidi SS Tashkandi M: A Simplified Method to Engineer CRISPR/Cas9-Mediated Geminivirus Resistance in Plants. *Methods Mol Biol.* 2019;2028:167–83. 10.1007/978-1-4939-9635-3_10 31228115

[58] Ma J Chen J Wang M: Disruption of *OsSEC3A* increases the content of salicylic acid and induces plant defense responses in rice. *J Exp Bot.* 2018;69(5):1051–64. 10.1093/jxb/erx458 29300985 PMC6018903

[59] Acevedo-Garcia J Spencer D Thieron H: *mlo*-based powdery mildew resistance in hexaploid bread wheat generated by a non-transgenic TILLING approach. *Plant Biotechnol J.* 2017;15(3):367–78. 10.1111/pbi.12631 27565953 PMC5316926

[60] Hummel AW Doyle EL Bogdanove AJ: Addition of transcription activator-like effector binding sites to a pathogen strain-specific rice bacterial blight resistance gene makes it effective against additional strains and against bacterial leaf streak. *New Phytol.* 2012;195(4):883–93. 10.1111/j.1469-8137.2012.04216.x 22747776

[61] Zeng X Tian D Gu K: Genetic engineering of the *Xa10* promoter for broad-spectrum and durable resistance to *Xanthomonas oryzae* pv. *oryzae*. *Plant Biotechnol J.* 2015;13(7):993–1001. 10.1111/pbi.12342 25644581

[62] Hutin M Césari S Chalvon V: Ectopic activation of the rice NLR heteropair RGA4/RGA5 confers resistance to bacterial blight and bacterial leaf streak diseases. *Plant J.* 2016;88(1):43–55. 10.1111/tpj.13231 27289079

[63] Shantharaj D Römer P Figueiredo JFL: An engineered promoter driving expression of a microbial avirulence gene confers recognition of TAL effectors and reduces growth of diverse *Xanthomonas* strains in citrus. *Mol Plant Pathol.* 2017;18(7):976–89. 10.1111/mpp.12454 27362693 PMC6638256

[64] Robaglia C Caranta C: Translation initiation factors: a weak link in plant RNA virus infection. *Trends Plant Sci.* 2006;11(1):40–5. 10.1016/j.tplants.2005.11.004 16343979

[65] Pyott DE Sheehan E Molnar A: Engineering of CRISPR/Cas9-mediated potyvirus resistance in transgene-free *Arabidopsis* plants. *Mol Plant Pathol.* 2016;17(8):1276–88. 10.1111/mpp.12417 27103354 PMC5026172

[66] Chandrasekaran J Brumin M Wolf D: Development of broad virus resistance in non-transgenic cucumber using CRISPR/Cas9 technology. *Mol Plant Pathol.* 2016;17(7):1140–53. 10.1111/mpp.12375 26808139 PMC6638350

[67] Gauffier C Lebaron C Moretti A: A TILLING approach to generate broad-spectrum resistance to potyviruses in tomato is hampered by *eIF4E* gene redundancy. *Plant J.* 2016;85(6):717–29. 10.1111/tpj.13136 26850324

[68] Bastet A Robaglia C Gallois JL: eIF4E Resistance: Natural Variation Should Guide Gene Editing. *Trends Plant Sci.* 2017;22(5):411–9. 10.1016/j.tplants.2017.01.008 28258958

[69] Zaidi SS Tashkandi M Mansoor S: Engineering Plant Immunity: Using CRISPR/Cas9 to Generate Virus Resistance. *Front Plant Sci.* 2016;7:1673. 10.3389/fpls.2016.01673 27877187 PMC5099147

[70] Baltes NJ Hummel AW Konecna E: Conferring resistance to geminiviruses with the CRISPR–Cas prokaryotic immune system. *Nat Plants.* 2015;1:15145 10.1038/nplants.2015.145 PMC8612103 34824864

[71] Ji X Zhang H Zhang Y: Establishing a CRISPR-Cas-like immune system conferring DNA virus resistance in plants. *Nat Plants.* 2015;1:15144. 10.1038/nplants.2015.144 27251395

[72] Iqbal Z Sattar MN Shafiq M: CRISPR/Cas9: A Tool to Circumscribe Cotton Leaf Curl Disease. *Front Plant Sci.* 2016;7:475. 10.3389/fpls.2016.00475 27148303 PMC4828465

[73] Moore JW Herrera-Foessel S Lan C: A recently evolved hexose transporter variant confers resistance to multiple pathogens in wheat. *Nat Genet.* 2015;47(12):1494–8. 10.1038/ng.3439 26551671

[74] Krattinger SG Lagudah ES Spielmeyer W: A putative ABC transporter confers durable resistance to multiple fungal pathogens in wheat. *Science.* 2009;323(5919):1360–3. 10.1126/science.1166453 19229000

[75] Milne RJ Dibley KE Schnippenkoetter W: The Wheat *Lr67* Gene from the Sugar Transport Protein 13 Family Confers Multipathogen Resistance in Barley. *Plant Physiol.* 2019;179(4):1285–97. 10.1104/pp.18.00945 30305371 PMC6446772

[76] Risk JM Selter LL Chauhan H: The wheat Lr34 gene provides resistance against multiple fungal pathogens in barley. *Plant Biotechnol J.* 2013;11(7):847–54. 10.1111/pbi.12077 23711079

[77] Schnippenkoetter W Lo C Liu G: The wheat Lr34 multipathogen resistance gene confers resistance to anthracnose and rust in sorghum. *Plant Biotechnol J.* 2017;15(11):1387–96. 10.1111/pbi.12723 28301718 PMC5633760

[78] Chauhan H Boni R Bucher R: The wheat resistance gene *Lr34* results in the constitutive induction of multiple defense pathways in transgenic barley. *Plant J.* 2015;84(1):202–15. 10.1111/tpj.13001 26315512

[79] Krattinger SG Sucher J Selter LL: The wheat durable, multipathogen resistance gene *Lr34* confers partial blast resistance in rice. *Plant Biotechnol J.* 2016;14(5):1261–8. 10.1111/pbi.12491 26471973 PMC11388880

[80] Krattinger SG Kang J Bräunlich S: Abscisic acid is a substrate of the ABC transporter encoded by the durable wheat disease resistance gene *Lr34*. *New Phytol.* 2019;223(2):853–66. 10.1111/nph.15815 30913300 PMC6618152

[81] Cardi T Varshney R: Cisgenesis and genome editing: Combining concepts and efforts for a smarter use of genetic resources in crop breeding. *Plant Breed.* 2016;135(2):139–47. 10.1111/pbr.12345

